# Murine Cytomegalovirus Exploits Olfaction To Enter New Hosts

**DOI:** 10.1128/mBio.00251-16

**Published:** 2016-04-26

**Authors:** Helen E. Farrell, Clara Lawler, Cindy S. E. Tan, Kate MacDonald, Kimberley Bruce, Michael Mach, Nick Davis-Poynter, Philip G. Stevenson

**Affiliations:** a School of Chemistry and Molecular Biosciences and Centre for Children's Health Research, University of Queensland, Brisbane, Australia; b Institut für Klinische und Molekulare Virologie, Friedrich-Alexander-Universität Erlangen-Nürnberg, Erlangen, Germany

## Abstract

Viruses transmit via the environmental and social interactions of their hosts. Herpesviruses have colonized mammals since their earliest origins, suggesting that they exploit ancient, common pathways. Cytomegaloviruses (CMVs) are assumed to enter new hosts orally, but no site has been identified. We show by live imaging that murine CMV (MCMV) infects nasally rather than orally, both after experimental virus uptake and during natural transmission. Replication-deficient virions revealed the primary target as olfactory neurons. Local, nasal replication by wild-type MCMV was not extensive, but there was rapid systemic spread, associated with macrophage infection. A long-term, transmissible infection was then maintained in the salivary glands. The viral m131/m129 chemokine homolog, which influences tropism, promoted salivary gland colonization after nasal entry but was not required for entry *per se*. The capacity of MCMV to transmit via olfaction, together with previous demonstrations of experimental olfactory infection by murid herpesvirus 4 (MuHV-4) and herpes simplex virus 1 (HSV-1), suggest that this is a common, conserved route of mammalian herpesvirus entry.

## IMPORTANCE

Cytomegaloviruses (CMVs) infect most mammals. Human CMV (HCMV) harms people with poor immune function and can damage the unborn fetus. It infects approximately 1% of live births. We lack a good vaccine. One problem is that how CMVs first enter new hosts remains unclear. Oral entry is often assumed, but the evidence is indirect, and no infection site is known. The difficulty of analyzing HCMV makes related animal viruses an important source of insights. Murine CMV (MCMV) infected not orally but nasally. Specifically, it targeted olfactory neurons. Viral transmission was also a nasal infection. Like HCMV, MCMV infected cells by binding to heparan, and olfactory surfaces display heparan to incoming viruses, whereas most other mucosal surfaces do not. These data establish a new understanding of CMV infections and a basis for infection control.

## INTRODUCTION

Cytomegaloviruses (CMVs) infect most mammals. Human CMV (HCMV) infection, although often inapparent, harms immunocompromised patients and unborn infants (1). The shedding of HCMV in saliva, milk, and urine is well documented, yet how virions first enter new hosts has lacked a clear answer. Oral entry is often assumed, but the evidence is indirect and open to other interpretations. Infection is acquired usually in childhood, through close contact with a virus carrier. Its incidence is highest in developing countries and in less privileged socioeconomic groups of developed countries. Early acquisition—with 1-year seroconversion rates close to 100%—is associated with promiscuous salivary exchange (2). In developed world neonatal units, breastfeeding from virus carriers increases infection rates (3). However, infants feeding regularly from carrier mothers show seroconversion rates of typically 60% at 1 year, implying that the infectivity of ingested milk is low; and for low-birth-weight infants within the first month of life, when they may be fed milk by nasogastric tube alone, infection is rare. Consequently, poor infection by ingested virus is hard to distinguish from a more efficient infection of accidentally contaminated sites such as the nose. Limited functional separation of the nasopharynx and oropharynx in breast-feeding infants (for example, nasal milk regurgitation is common) further warns against inferring oral entry without identifying the infected cells.

A conceptual problem with gastrointestinal infection is that acid inactivates HCMV (4) and should do so even in preterm infants (5), as should lipid emulsification by bile: viruses known to infect orally have nonenveloped, acid-resistant virions. Oropharyngeal infection would avoid these insults, but saliva constantly washes the oral contents to the stomach. Viral evolution is driven by transmission efficiency, and it is hard to see how oropharyngeal infection by milk or saliva could be efficient in so short a time.

While HCMV host entry is hard to track, the ubiquity of mammalian CMV implies that their parasitism long preceded human emergence and that animal and human CMVs should behave similarly. Orally inoculated rhesus CMV can infect macaques (6). However, experimental inoculation involves giving high virus doses to sedated animals, making respiratory contamination hard to exclude, and again, no oral infection site has been identified. Murine CMV (MCMV) transmission via breast milk is reported (7), but infected gastrointestinal cells were not found after oral inoculation (8). Inhaled virions can infect the lungs (9), yet delivery here depends on inoculation under anesthesia (10). Another report identified MCMV infection of olfactory epithelial cells and nasal-associated lymphoid tissue 5 days after a high-volume combined oral/nasal inoculation (11). Olfactory infection has obvious relevance to earlier descriptions of olfactory entry by murid herpesvirus 4 (MuHV-4) (12) and herpes simplex virus 1 (HSV-1) (13). However, infection at 5 days cannot be interpreted as host entry: by this time after inhalation, HSV-1 has spread via the trigeminal ganglia to the facial skin (13), and inhaled MuHV-4 colonizes the nasal-associated lymphoid tissue only as a secondary site (14). To identify primary infection, it is necessary to look early, ideally with a virus limited to a single infection cycle.

A criticism of any experimental inoculation is that it might deliver virus to a site not normally reached. Large inoculation volumes may also create artifacts, as viruses normally transmit in small volumes (15). To minimize experimental artifacts, we have tracked host entry after spontaneous small-volume virus ingestion or inhalation without anesthesia (10). However, the gold standard remains a demonstration of transmission. This is likely to be a low-dose infection and to present well after virus uptake. We used sensitive live imaging of virus-expressed luciferase to reduce the time lag between virus uptake and detectable infection. We found that MCMV acquired spontaneously by alert mice infects nasally rather than orally, both when presented as a low-volume droplet and during natural transmission. The primary target was olfactory neurons, implying that MCMV exploits olfaction to enter new hosts.

## RESULTS

### MCMV infects nasally, not orally.

**FIG 1:**
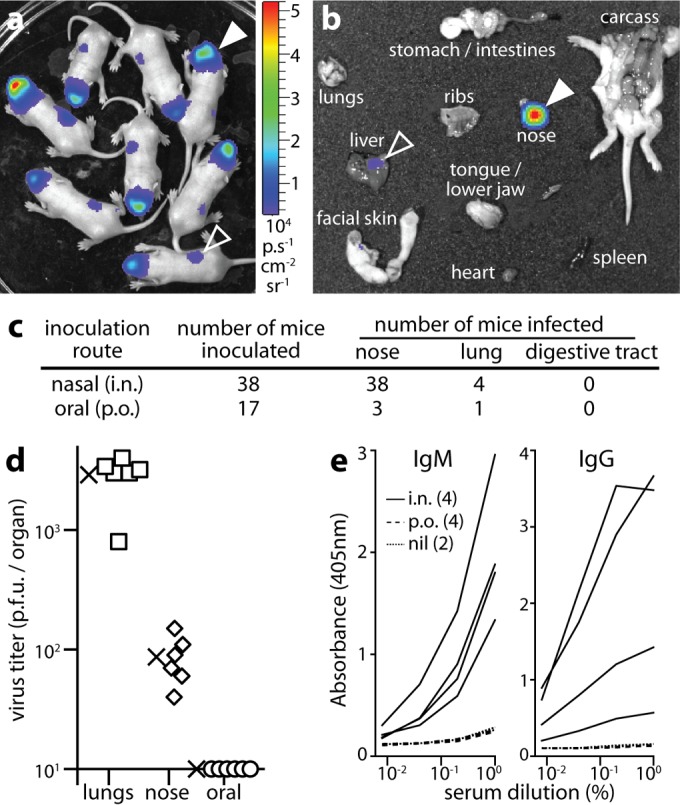
MCMV infects pups nasally. (a) BALB/c pups imaged 2 days after i.n. MCMV-LUC (3 × 10^4^ PFU) showed nasal luciferase signals (filled arrowhead). Liver signals (open arrowhead) were nonspecific. The images shown are representative of the images for 26 mice. The heatmap color bar shows 10^4^ photons/s/cm^2^/sr. (b) Dissection at day 2 confirmed nasal infection (filled arrowhead) and weak liver signals (open arrowhead). The images shown are representative of the images for 12 mice. (c) MCMV was consistently more infectious i.n. than p.o., and MCMV infected noses more commonly than it infected lungs (both *P* < 10^−4^ by Fisher's exact test). At day 4 postinoculation, no mice had oropharyngeal or gastrointestinal signals, and all mice infected p.o. were infected in the respiratory tract. Results were pooled from three independent experiments. (d) At 4 days after i.n. inoculation, noses were plaque assayed for infectious virus and compared with the lungs of mice that had luciferase-positive lungs (due to inoculum aspiration), and the oropharynges of mice given MCMV p.o. (oral). The means are indicated by × symbols. The other symbols represent the values for individual mice. The *x* axis shows the limit of assay sensitivity. (e) At 1 month postinoculation, mice were assayed for MCMV-specific serum IgG and IgM by ELISA. The mice were given MCMV i.n. (with no evidence of lung infection; *n* = 4), MCMV p.o. (with no evidence of respiratory infection; *n* = 4), or no virus (nil; *n* = 2).

Most CMV infections occur early in life, via inhaled or ingested virions. Thus, to identify viable entry routes, we gave alert, 2- to 5-day-old BALB/c mice luciferase-positive MCMV (MCMV-LUC) (3 × 10^4^ PFU in 1 µl) either nasally (i.n.) or orally (per os [p.o.]) and tracked infection by live imaging (Fig. 1). All i.n.-inoculated mice showed nasal luciferase signals (Fig. 1a, filled arrowhead) that were confirmed by dissection (Fig. 1b, filled arrowhead). Weak signals were also seen in livers at up to 2 weeks of age (Fig. 1a and b, open arrowheads), but these were unrelated to infection, as they also occurred in naive mice, and unlike nasal signals were not abolished by preincubating the virus with sera from immune mice (0.5-µl serum per 3 × 10^4^ PFU virus, 2 h at 23°C).

Abdominal signals were seen rarely (<1/20 mice). Dissections invariably showed that these were from the gut contents rather than the gut itself. They appeared to reflect accidental gut injection by intraperitoneal (i.p.) luciferin: the 4/17 p.o.-inoculated mice that became luciferase positive (Fig. 1c) all had respiratory, rather than gastrointestinal, signals. Plaque assays (Fig. 1d) confirmed nasal infection by i.n. MCMV and a lack of oral infection by p.o. MCMV. Enzyme-linked immunosorbent assays (ELISAs) of virus-specific serum IgM and IgG after 1 month (Fig. 1e) further confirmed infection by i.n., but not p.o., MCMV. Therefore, primary infection was limited to the respiratory tract.

### MCMV targets the upper respiratory tract.

Even 1 µl is a large volume for pups to inhale, so occasional inoculum aspiration into the lungs (Fig. 1c) was unsurprising. Early (day 2) lung infections [mean ± standard deviation (SD) for six mice = (415 ± 224) × 10^4^ photons/s/cm^2^/sr (p/s/cm^2^/sr)] gave stronger signals than nasal infections [(18.7 ± 8.9) × 10^4^] (*P* < 10^−4^) and yielded more virus (Fig. 1d). Therefore, virus reaching the lung was easier to detect than virus reaching the nose. However, at day 2, >80% of pups showed only nasal signals. Lung signals occurred in approximately 50% of these pups after day 5, but these later lung signals were weaker than the nasal signals and presumably reflected a primary upper respiratory tract infection spreading to the lower respiratory tract.

### Nasal infection disseminates to the salivary glands.

**FIG 2:**
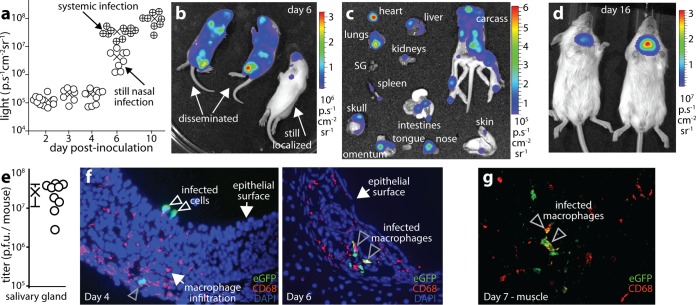
MCMV spreads from the nose to the salivary glands. (a) BALB/c pups given MCMV-LUC i.n. (3 × 10^4^ PFU) were tracked by live imaging. On days 2 to 4, luciferase signals were only nasal; on day 6, some mice (circles containing + symbols) showed systemic infection spread and had significantly stronger signals than those with only nasal infection (open symbols) (*P* < 0.01); by day 10, infection had spread in all mice. The means are indicated by × symbols. The circles represent the values for individual mice. (b) Day 6 images show examples of localized and disseminated infections. (c) Dissection of a mouse with disseminated infection at day 6 shows involvement of multiple organs. The images shown are representative of the images for six mice. SG, salivary gland. (d) By day 16, live image signals were confined to the salivary gland. The images shown are representative of the images for 12 mice. (e) Salivary glands of mice given K181 MCMV i.n. (3 × 10^4^ PFU) 1 month earlier were plaque assayed for infectious virus. The circles represent values for individual mice. The mean (×) ± standard error of the mean (SEM) (error bars) are shown. (f) At day 4 after i.n. inoculation of eGFP^+^ MCMV (3 × 10^4^ PFU), the nasal (olfactory) epithelium showed CD68^+^ macrophage infiltration at sites of infection. The gray arrowhead indicates an infected CD68^+^ cell. At day 6 postinoculation, infected macrophages were more obvious and more widespread. The images shown are representative of the images for eight mice. (g) Pups were given MCMV-LUC i.n. (3 × 10^4^ PFU). At the early stage of systemic spread (7 days postinoculation), luciferase-positive systemic sites were sectioned and stained to identify infected cells. Most were macrophages. The image shows a section of muscle from the chest wall. The image shown is representative of the images for six mice.

MCMV-LUC gave substantially lower peak nasal signals [mean ± SD for 12 mice = (3.3 ± 0.9) × 10^6^ p/s/cm^2^/sr] than luciferase-positive MuHV-4 or HSV-1 (>10^8^ p/s/cm^2^/sr) (11, 12). However, MCMV signals increased dramatically when infection spread (Fig. 2a), with a marked difference at day 6 between mice that still had local infections and mice whose infection had spread (Fig. 2b). (Minimally invasive infections spread less synchronously than invasive infections, because additional tissue barriers must be crossed.) Dissecting day 6 mice that exhibited infection spread by live imaging (Fig. 2c) revealed signals from many organs, including heart, lungs, liver, spleen, muscle, and subcutaneous fat. Blood was negative (data not shown). By day 10, infection had spread in all mice (Fig. 2a). They flourished nonetheless; only primary lung infections retarded growth. By day 16, luciferase signals were restricted to the submandibular salivary glands (Fig. 2d), where they persisted for more than a month with high titers of recoverable virus (Fig. 2e). Therefore, nasal entry reproduced the natural pattern of MCMV persistence in the salivary glands (16).

MCMV inoculated i.p. establishes a monocyte-associated viremia (17). Monocytes or macrophages also appeared to spread MCMV from the olfactory epithelium (Fig. 2f): at day 4, cells expressing the pan-macrophage marker CD68 (18) infiltrated infected neuroepithelial sites; at day 6, infected CD68^+^ cells were evident in the epithelium; and at day 7, infected CD68^+^ cells appeared in distant luciferase-positive sites such as muscle and fat.

### The m131/m129 viral tropism determinant is redundant for nasal infection.

**FIG 3:**
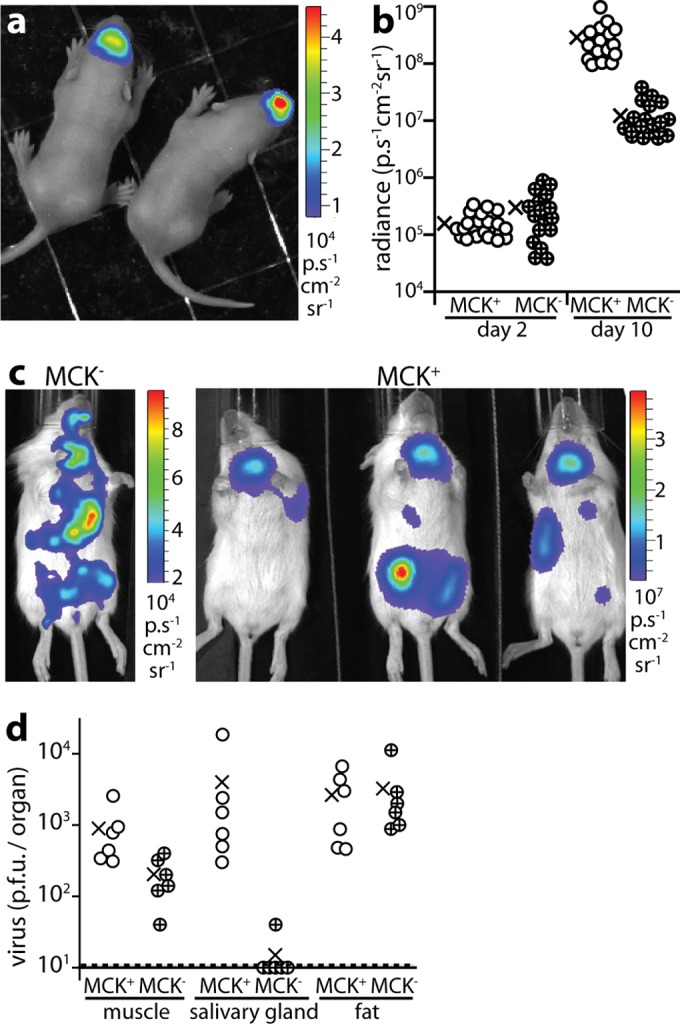
MCK2 is redundant for MCMV nasal infection but not for subsequent spread. (a) BALB/c pups imaged 2 days after i.n. MCK2^−^ MCMV-LUC (3 × 10^4^ PFU). The image shown is representative of the images for 16 mice. (b) Live images showed no i.n. infection defect of MCK2^−^ MCMV-LUC at day 2 but significantly less disseminated signal than MCK2^+^ infected controls at day 10 (*P* < 0.001). The circles represent values for individual mice. The means are indicated by × symbols. (c) Live imaging after day 10 showed dissemination but little salivary gland infection by i.n. MCK2^−^ MCMV compared to MCK2^+^. Note the different image scales. The images shown are representative of the images for six mice. (d) Plaque assays 10 days after i.n. inoculation showed significantly less MCK2^−^ MCMV than MCK2^+^ MCMV in salivary glands (*P* < 0.01) but not in muscle (*P* = 0.08) or fat (*P* = 0.8). The circles represent values for individual mice. The means are indicated by × symbols.

The MCMV m131/m129 encodes a chemokine homolog (MCK2) (19) that promotes macrophage infection (20). MCK2-negative (MCK2^−^) MCMV is described as poorly infecting the lungs (8). MCK2^−^ MCMV-LUC showed no defect in entering noses (Fig. 3a and b) but spread poorly: day 10 MCK2^−^ luciferase signals (mean ± SD for five mice = 393 ± 217 p/s/cm^2^/sr) were significantly lower than MCK2^+^ (mean ± SD for six mice = 9,739 ± 5,144  p/s/cm^2^/sr) (*P* < 0.001) (Fig. 3c). Plaque assays (Fig. 3d) confirmed poor salivary gland infection. Muscle and fat infections were not significantly impaired.

### MCMV nasally infects adult mice.

**FIG 4:**
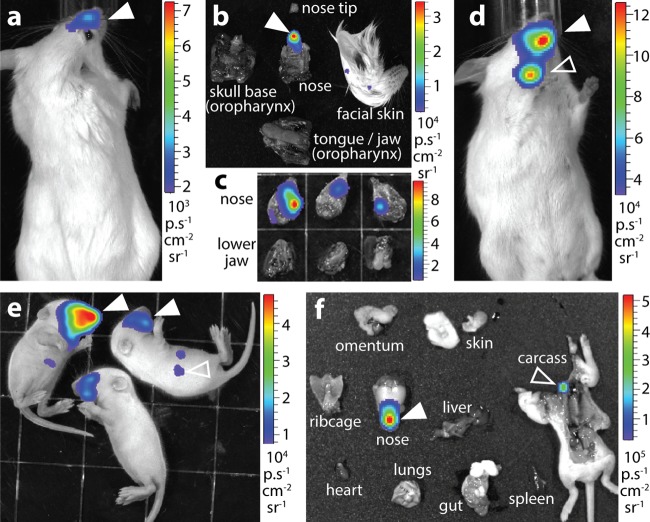
Adult mice also show nasal MCMV host entry. (a) Live image of an adult BALB/c mouse 4 days after i.n. MCMV-LUC (10^5^ PFU). The image shown is representative of the images for eight mice. (b) *Ex vivo* imaging confirmed nasal infection (filled arrow) and not oropharyngeal infection. The images shown are representative of the images for eight mice. (c) *Ex vivo* imaging of C57BL/6 mice 4 days after i.n. MCMV-LUC (10^5^ PFU) showed nasal and not oropharyngeal signals. The images shown are representative of the images for six mice. (d) Live image of an adult BALB/c mouse 4 days after i.n. M78-LUC MCMV (10^5^ PFU), showing nasal (filled arrowhead) and cervical lymph node (open arrowhead) signals. The image shown is representative of the images for six mice. (e) Live imaging of BALB/c pups 2 days after i.n. M78-LUC (3 × 10^4^ PFU) showing nasal signals (filled arrowheads) and background liver signals (open arrowhead). The image shown is representative of the images for 12 mice. (f) *Ex vivo* imaging of a representative BALB/c pup (*n* = 12) 2 days after i.n. M78-LUC (3 × 10^4^ PFU) showing a strong nasal signal (filled arrowhead) and a weak cervical lymph node signal (open arrowhead). The images shown are representative of the images for six mice.

Most viruses replicate better in neonates than in adults, and <20% of adult mice inhaling MCMV-LUC (10^5^ PFU) had infections detectable by live imaging (Fig. 4a). However, dissection, which increases luciferase detection sensitivity approximately 10-fold, consistently revealed infection in the noses of BALB/c (Fig. 4b) and C57BL/6 (Fig. 4c) adult mice. Moreover, MCMV K181 strain expressing luciferase from the M78 promoter (M78-LUC) (Fig. 4d, filled arrowhead) gave signals detectable by live imaging in all 12 BALB/c adults (10^5^ PFU i.n.). Spread to the superficial cervical lymph nodes was also evident (Fig. 4d, open arrowhead). M78-LUC targeted the noses and not the gastrointestinal tracts or oropharynges of all 12 i.n.-inoculated pups (Fig. 4e and f). M78-LUC inoculated p.o. infected neither pups (0/6 mice were luciferase positive, 3 × 10^4^ PFU) nor adults (0/6 mice, 10^5^ PFU). Nor did i.n. M78-LUC give lung signals in adults (0/6 mice, 10^5^ PFU) unless they were infected under anesthesia (6/6 mice). Therefore, upper respiratory tract infection was consistent across MCMV strains, mouse strains, and mouse ages.

### Incoming virions target olfactory neurons.

**FIG 5:**
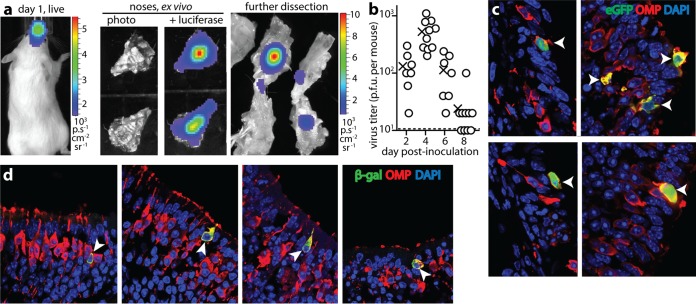
Identification of the nasal entry target. (a) Dissecting live image mice 1 day after i.n. M78-LUC revealed nasal infection. Further dissection localized signals to the nasal sinuses. The images shown are representative of the images for six mice. (b) Plaque assay of C57BL/6 pup noses after i.n. MCMV-GR (3 × 10^4^ PFU). The circles represent values for individual mice. The means are indicated by × symbols. (c) Nose sections of C57BL/6 pups given MCMV-GR i.n. (10^5^ PFU) 3 days earlier were stained for viral eGFP expression and olfactory marker protein (OMP) after 3 days. Nuclei were stained with DAPI. All eGFP^+^ cells were OMP^+^ (white arrowheads). The image shown is representative of the images for 12 mice. (d) Noses of pups given gL^−^ MCMV i.n. (10^5^ PFU) were stained 1 day later for viral β-galactosidase (β-gal) and OMP. All β-galactosidase-positive cells were OMP^+^ (white arrowheads).The images shown are representative of the images for six mice.

Dissecting pup noses 3 days after M78-LUC inhalation localized luciferase signals to the nasal sinuses (Fig. 5a). We did not observe early (day 1 to day 3) luciferase signals from the nasal-associated lymphoid tissue with either MCMV-LUC (HCMV IE1 promoter) or M78-LUC (early lytic M78 promoter). To label infected cells for immunostaining on tissue sections, we used eGFP^+^ (enhanced green fluorescent protein-positive) MCMV (MCMV-GR), giving it to C57BL/6 mice, as BALB/c mice recognize an H2K^d^-restricted epitope in eGFP (21), which might lead to virus attenuation if eGFP is expressed in latency. As with MCMV-LUC, acutely infected noses had only low virus titers (Fig. 5b). Nonetheless, eGFP^+^ cells were evident on tissue sections (Fig. 5c). All were olfactory neurons (*n* = 12 mice), based on residence in the olfactory epithelium and expression of olfactory marker protein, a cytoplasmic signal transduction protein common to all vertebrates and specific to olfactory sensory neurons (22). To identify formally the cells first infected, we inoculated mice with MCMV lacking its essential glycoprotein L (gL) (Fig. 5d). Virions were pseudotyped gL positive (gL^+^) by growth in gL^+^ cells (23) and so could infect just once. Staining for viral β-galactosidase revealed infection exclusively in olfactory neurons (*n* = 6 mice). Infection was not seen in vomeronasal neurons (data not shown). Therefore, MCMV targeted neurons of the main olfactory epithelium.

The olfactory epithelium comprises chiefly neurons and sustentacular cells (24, 25). The sustentacular cell nuclei form a layer above the neurons, but each neuron projects a terminal dendrite apically between the sustentacular cells, with which it forms tight junctions, and from each dendrite sprout long, nonmotile cilia. These cilia are embedded in olfactory mucus and form the apical epithelial surface. Thus, the neuronal cilia provide a bridge across the olfactory mucus, with retrograde transport potentially moving attached virions along cilia to the endocytic pit of the terminal dendrite. The apical sustentacular surface consists of shorter microvilli, which extend only into the basal mucus. Inhaled MuHV-4 infects sustentacular cells directly (25), suggesting that their microvilli can capture virions from neuronal cilia. MCMV infected sustentacular cells at day 3 (see Fig. S1 in the supplemental material), but not at day 1 nor if gL disruption prevented spread. Therefore, for MCMV, sustentacular cells were a secondary target. Similarly, by day 5, infected macrophages were evident in subepithelial sites (Fig. S1), including around the nasal-associated lymphoid tissue, but day 1 and gL^−^ infections were confined to olfactory neurons.

Most mammalian herpesviruses bind to heparan. While transformed epithelial cells and fibroblasts express heparan indiscriminately, differentiated epithelia do so only basolaterally (26). The olfactory epithelium is a notable exception (25). Like MuHV-4 (27), HSV-1 (28), and HCMV (29), MCMV shows heparan-dependent infection (see Fig. S2 in the supplemental material). Therefore, while the *in vivo* distribution of MCMV protein receptors is unknown, viral heparan dependence and apical olfactory heparan expression provided a molecular explanation for neuroepithelial virion capture.

### MCMV transmits nasally between pups.

**FIG 6:**
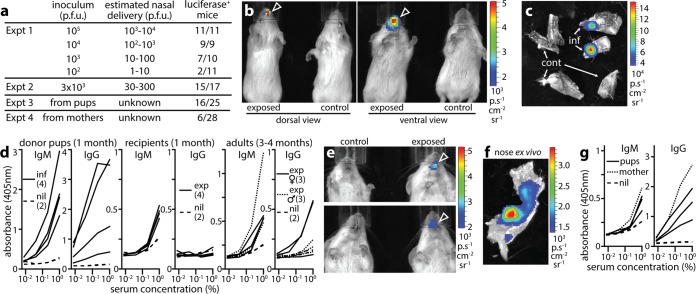
MCMV transmission is a nasal infection. (a) Experiment 1 (Expt 1) established a minimum *in vivo* infectious dose for giving M78-LUC i.n. in 1 µl to BALB/c pups (10^3^ PFU). Experiment 2 tracked infection after giving the minimum infectious dose i.n. three times per mouse. Experiment 3 tracked M78-LUC acquisition by naive recipient pups cocaged with infected donor pups. Experiment 4 tracked M78-LUC acquisition by pups from infected parents. (b) Live images of luciferase expression from a mouse acquiring M78-LUC infection in experiment 3 (exposed) compared with an unexposed control mouse. See also Fig. S3 in the supplemental material. (c) *Ex vivo* images of luciferase expression in the nose of an infected (inf) mouse from experiment 3 (*n* = 1), with control (cont) unexposed noses shown for comparison (*n* = 2). Each nose was split sagitally to reveal the septum and so increase imaging sensitivity. (d) ELISA of MCMV-specific serum IgM and IgG from infected (inf) donor pups, exposed (exp) recipient pups, and exposed male and female parents in experiment 3. Age-matched, naive controls (nil) are also shown. The duration of cocaging with MCMV-infected pups is shown. (e) Luciferase signals in exposed (arrowheads) and unexposed control pups of experiment 4. See also Fig. S4 in the supplemental material. Although the signal in the top panel looks more oral than nasal, this is due to nasal signal being seen best through the mouth because of less light attenuation along this line of sight. Dissection (see panel f) confirmed that the signal was nasal in origin. (f) Dissection of the mouse in the top panel in panel e showing luciferase expression in the nasal sinuses. Other tissues were negative. (g) ELISA of MCMV-specific serum IgM and IgG from exposed pups in experiment 4 (*n* = 3), their mother (*n* = 1), and control, unexposed pups (nil) (*n* = 2).

Minimally invasive inoculations proved that nasal infection is possible; to establish whether it is physiologically relevant, we tracked MCMV transmission. Analogy with HCMV suggested that this would be a low-dose infection prior to weaning. To establish how low-dose i.n. infection might present, we inoculated alert pups with various doses of M78-LUC in 1 µl and imaged dissected tissues for luciferase expression at day 10 (Fig. 6a, experiment 1 [Expt 1]). The minimum infectious dose was 10^3^ PFU. This did not mean that *in vivo* infection was inefficient: adult mice retain <1 µl in their nasal sinuses (10), and pups show a similarly limited nasal retention of inhaled dyes (data not shown). Thus, <10^2^, possibly <10 PFU, of the inhaled 10^3^ PFU was retained.

By using live imaging, we tracked pups administered with three sequential minimum infectious doses (equivalent to 3 × 10^3^ PFU; Fig. 6a, Expt 3). Whereas 10^5^ PFU gave signals after 2 to 5 days, 3 × 10^3^ PFU did so after 8 to 23 days. Of 15 positive mice, 8 presented with nasal infection and 7 with disseminated infection, involving variously the liver, lungs, salivary glands, and nose. Figure S3 in the supplemental material shows examples. Thus, while all mice were infected nasally, primary virus replication was sometimes below the limit of live image detection. This illustrates an important point about experimental models: inoculations are often chosen for their capacity to establish large primary infections, but virus evolution is driven by transmission, and herpesviruses transmit by long-term shedding, so extensive primary replication may confer no selective advantage and may not be a prominent feature of natural infection. Nasal replication by i.n.-inoculated MCMV replication was never extensive. Moreover, live imaging showed only moderate sensitivity, detecting only 2/9 of the low-dose infections detected by day 10 *ex vivo* imaging in experiment 1 in Fig. 6a. However, to catch early events, this insensitivity was outweighed by a capacity for serial monitoring, as transmission was sporadic and asynchronous between mice.

Children acquire HCMV from acutely infected peers and carrier mothers. To transmit MCMV between pups (Fig. 6a, Expt 3), we bred trios of two female BALB/c mice plus one male BALB/c mouse. The females became pregnant typically a week apart. We infected the first (donor) litter i.n. at 1 to 3 days (3 × 10^4^ PFU in 1 µl). Any excess inoculum was cleaned from the external nares, and the pups were returned to their cage. After 1 week, they had strong salivary gland signals that were then maintained. The subsequent (recipient) litter from the other female was then monitored by live imaging every 2 or 3 days from 1 to 4 weeks of age. Any luciferase-positive pups, and all pups after 4 weeks, were killed for *ex vivo* analysis. In cages with dust-free bedding (Enviro-dri; Shepherd), the mice made shallow nests in which they spent little time, and 0/21 recipient pups were luciferase positive. With cardboard containers (Shepherd Shack; Shepherd) and additional bedding (Alpha Pad; Shepherd), the mice spent most of their time in enclosed nests, and 16/25 recipients had luciferase-positive noses: 7 by live imaging and 9 by *ex vivo* imaging at 4 weeks. Of the live image-positive mice, four had just nasal infection (Fig. 6b and c; see Fig. S3 in supplemental material), and three had disseminated infection including the nose. None had oral or gastrointestinal infection. Infectious virus was recovered by plaque assay from 8/16 luciferase-positive recipient noses (mean ± SD of 15.0 ± 7.6 PFU/nose).

PCR amplification of viral DNA detected nasal infection less sensitively than plaque assay: for equivalent-sized samples, PCR was 150 times more sensitive, but it assayed only 100 ng of 500-µg total DNA, whereas whole-nose homogenates could be plaque assayed. Thus, positive-control noses (3 × 10^4^ PFU i.n.) yielded 35.1 ± 15.8 PFU per PCR-detected viral genome (mean ± SD for six mice), and PCR detected viral genomes in only 2/12 plaque-positive recipient noses. Serology (Fig. 6d) showed virus-specific serum IgG and IgM in donor mice after 4 weeks, and virus-specific serum IgM in all luciferase-positive recipients. Virus-specific IgM was also detected in two luciferase-negative recipients. After 3 to 4 months (two breeding cycles), 9/12 parental mice had MCMV-specific serum IgM or IgG, consistent with infection acquisition from their pups.

### MCMV transmits nasally to pups from carrier adults.

To transmit MCMV from mothers to pups, we infected adult breeding trios i.n. (10^5^ PFU in 5 µl without anesthesia) (Fig. 6a, Expt 4). This caused no illness. Females typically became pregnant 3 to 4 weeks later, with normal litter sizes and healthy pups. Adult salivary gland infection was detectable by live imaging during pregnancy and lactation—although with 100-fold-lower signals than the donor pups in experiment 3 in Fig. 6a (see Fig. S4 in the supplemental material). We did not observe convincing mammary gland signals. Without enclosed nests, 0/25 pups showed luciferase signals; with them, 6/28 pups showed signals. Thus again, transmission depended on close cohabitation. We did not formally compare housing conditions, aiming simply to transmit MCMV in a setting that allowed us to track its route. Nonetheless, it was clear that suckling alone transmitted infection poorly.

All recipient pup luciferase signals were nasal: four by live imaging and two by *ex vivo* imaging at day 28 (Fig. 6e and f; see Fig. S4 in the supplemental material). The signals were weak, but they were absent from other organs and from nonexposed pups. Dissection showed distributions consistent with virus inhalation, while oropharyngeal and gastrointestinal signals were lacking. Saliva seemed the most likely source of transmission, as most adult signals were salivary, and nest building was not required for breastfeeding yet promoted transmission. In contrast to Fig. 6a (Expt 3), recipients did not show disseminated luciferase signals, possibly because maternal antibody limited acute virus spread, nor did they yield infectious virus, but they made virus-specific IgM (Fig. 6g). MCMV-specific serum IgM was also detected in 3 of the 22 luciferase-negative mice of Fig. 6a (Expt 4). The antibody response to naturally transmitted virus clearly developed slower than that to experimental infection, presumably because the infectious dose was less. The antibody response to HCMV also develops slowly (30), as does that to inhaled MuHV-4 (31), suggesting that rapid responses to experimentally injected viruses might reflect dose and inoculation route more than species differences.

**FIG 7:**
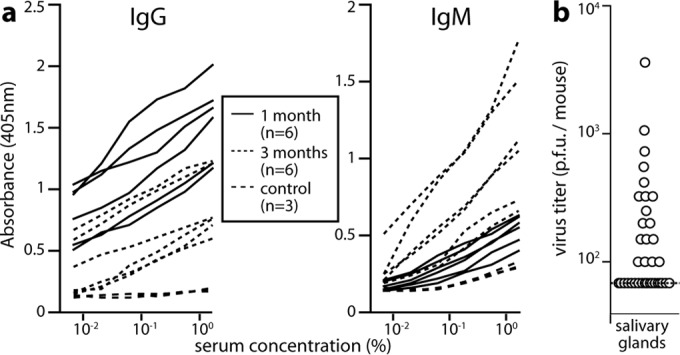
Longer-term MCMV transmission between cohoused BALB/c mice. (a) Alert male and female adult mice were infected nasally with K181 MCMV (10^5^ PFU in 5 µl), then mated as trios with access to nesting materials. Pups were test bled and weaned at 4 weeks, maintained with sex-matched littermates for a further 2 months, and then tested for MCMV infection by ELISA of virus-specific serum IgG and IgM and plaque assay of homogenized salivary glands. Serological results at 1 month and 3 months plus serological results of age-matched controls are shown. Of 36 mice, 26 mice were IgM^+^. IgG serology was considered less reliable because of the difficulty of distinguishing new responses from residual maternal IgG. Examples of positive responses are shown. (b) The same mice as in panel a were tested for infection by plaque assay of salivary glands at 3 months of age. Of 36 mice, 18 mice had recoverable virus. The circles represent titers for individuals. The dashed line shows the limit of assay sensitivity.

Our aim in tracking transmission was to identify its route rather than its efficiency. Thus, experiments were short-term, with exposure for 1 month. Reliable HCMV transmission requires close contact over several months (3). Therefore, to look for equivalent MCMV transmission, we allowed more time for infection to be passed on and to manifest. We infected adult BALB/c mice i.n. with wild-type K181 MCMV (10^5^ PFU in 5 µl) rather than M78-LUC, as we were not tracking the infection route and allowed breeding as before. The pups were weaned and segregated by sex at 4 weeks of age, but they continued to live as groups in nests. At 3 months of age, they were tested for virus-specific serum IgM by ELISA and for salivary gland infection by plaque assay (Fig. 7). Of 36 mice, 26 were IgM^+^ and 18 had recoverable virus. Therefore, i.n. MCMV established a chronic infection transmissible to the next generation.

## DISCUSSION

How mammalian herpesviruses enter new hosts has been unclear. This reflects the difficulty of studying pathogens whose acquisition, persistence, and transmission are often asymptomatic. Oral entry is hypothesized for most human herpesviruses. However, the supporting data are indirect, and no convincing entry site has been identified. Respiratory transmission is assumed for varicella-zoster virus (32) and some veterinary herpesviruses (33) but again with no known entry site. Experimental low-volume inoculations of alert mice with murid herpesvirus 4 (MuHV-4) and herpes simplex virus 1 (HSV-1) (12, 13) have demonstrated the olfactory epithelium as an entry site. Because HSV-1 does not transmit between mice and only sexual transmission is known for MuHV-4 (34), whose natural host is *Apodemus flavicollis* (35), it has been possible to conclude that olfactory infection follows if these alpha- and gammaherpesviruses are inhaled, but not that naturally shed virions are inhaled. We showed here that MCMV, a betaherpesvirus, targets olfactory neurons to enter its natural host and that upper respiratory tract infection is the predominant mode of transmission between captive mice.

Most differentiated epithelia express only basolateral heparan (26). The olfactory epithelium also expresses heparan on its apical surface, which comprises neuronal cilia embedded in mucus (24, 25). Olfactory infection by MuHV-4 depends on its heparan binding proteins (25), and the heparan dependence of MCMV and HSV-1 is consistent with heparan binding also driving their olfactory infections. Having crossed the olfactory mucus on neuronal cilia, virions must enter the terminal neuronal dendrite. This almost certainly involves additional receptor binding; for example, the HSV-1 receptor nectin 1 localizes to tight junctions between neuronal dendrites and sustentacular cells (13). However, the high valency of heparan binding makes the release of captured virions unlikely. Thus, while events after heparan binding can limit the infection of cell lines, a virus that failed to infect after binding *in vivo* would struggle to evolve. Therefore, we propose that heparan binding is the key decision point of olfactory targeting and herpesvirus host entry.

After olfactory entry, MCMV, MuHV-4, and HSV-1 disseminate along distinct paths: MuHV-4 uses dendritic cells to reach draining lymph node B cells (36), HSV-1 reaches the trigeminal ganglia via its nasal branches (13), and MCMV-infected immigrant myeloid cells reach the salivary glands. None showed luciferase expression in the olfactory bulbs. Indeed, centripetal spread along olfactory neurons seems to be rare for any virus unless the dose is high or the host is immunocompromised, possibly reflecting strong antiviral responses and a capacity for neuronal apoptosis and replacement (37). Thus, for each virus, olfactory entry is entirely compatible with the natural history of infection.

An argument against respiratory herpesvirus infection has been that transmission requires close contact. However, the proximity of the nasopharynx and oropharynx make their infections hard to distinguish epidemiologically, and a need for close contact identifies only low virus shedding—MCMV was far from contagious despite nasal entry. Another argument has been that acute clinical lesions are often oral. However, interpreting these lesions as host entry is problematic because herpesviruses spread systemically and reemerge in new sites. For example, varicella-zoster virus host entry is clinically silent, and the presenting skin vesicles of varicella reveal host exit. Epstein-Barr virus (EBV) and HSV oropharyngeal lesions seem similarly to follow spread to the latency reservoir, as prompt therapy does not reduce long-term infection (38, 39). In mice, nasal MuHV-4 reemerges in submucosal lymphoid tissue (14) and the genital tract (34), nasal HSV-1 reemerges in perioral skin lesions after colonizing the trigeminal ganglia (13), and nasal MCMV reemerges in the salivary gland.

Experimental rhesus CMV (RhCMV) and rhesus lymphocryptovirus (RhLCV) infections by oral inoculation seemingly support the idea of oral entry. However, it remains necessary to identify the cells infected. MCMV, MuHV-4, or HSV-1 can all infect anesthestized mice after oral inoculation, but luciferase imaging invariably reveals a spillover infection of the upper or lower respiratory tract. Extrapolating from nonhuman to human biology always provokes controversy. However, MCMV diverged from MuHV-4 200 million years (Ma) ago and from HSV-1 400 Ma ago (40), so shared olfactory entry implies that this predates primate/rodent divergence (70 Ma ago). The fact that herpesviruses differ most in genes interacting with diverse functions in the host, such as immune responses, suggests that their evolution is driven by host diversification, with rapid viral adaptation then restoring the status quo. Hence, CD8^+^ T cell evasion shows mechanistic diversity but conserved function (41). What host diversification would drive a change in entry route is unclear, and multiple viral glycoprotein changes would be required. Further, if oral herpesvirus entry were possible, its absence in mice would be puzzling, as they consume >10% of their body weight daily and derive key vitamins from coprophagia. Rodents devote more of their brains to olfactory discrimination, but primate olfaction remains sensitive, and although it is less important socially, it is evident between mothers and infants and between sexual partners, both common settings for herpesvirus transmission. Thus, we propose that olfactory transmission exploiting close contact behaviors is an ancestral characteristic of herpesviruses that is maintained in diverse hosts. An important prediction is that rhesus CMV (and rhesus lymphocryptovirus) will infect macaques i.n. Recognizing the possibility of olfactory transmission provides a basis for reducing the incidence of CMV infections, and through defining its molecular components, for delivering preventative vaccines.

## MATERIALS AND METHODS

### Mice.

MCMV was given i.n. by pipetting it onto the nares in 5 µl (adults) or 1 µl (pups) under light restraint without anesthesia and p.o. by pipetting the same volumes into the mouth. Nasal inoculation was atraumatic and did not involve touching the nose with the pipette tip. For lung inoculation, mice were anesthetized with isoflurane and MCMV was given i.n. in 30 µl. For imaging, mice were given 1 mg (pups) or 2 mg (adults) d-luciferin i.p., anesthetized with isoflurane, and scanned with a Xenogen IVIS-200 imaging system. Statistical analysis was performed by using Student’s two-tailed unpaired *t* test unless stated otherwise. Experiments were approved by the University of Queensland Animal Ethics Committee (project 301/13) in accordance with National Health and Medical Research Council guidelines.

### Cells and viruses.

K181 MCMV with an HCMV IE1 promoter-driven LacZ cassette disrupting gL was grown in gL^+^ NIH 3T3 cells (22). Other viruses were grown in NIH 3T3 cells. MCK2^−^ MCMV-LUC has an HCMV IE1 promoter-driven luciferase in m157 of strain Smith, and an inactivating frameshift in MCK2 (42). MCMV-LUC was made the same way after MCK2 repair (43). M78-LUC was made from strain K181 (MCK2^+^) by fusing luciferase to the M78 C terminus via an autocatalytic, *cis*-acting hydrolase element (44). MCMV-GR has HCMV IE1 promoter-driven floxed eGFP upstream of tdTomato, in m157 of strain K181 (23). In cre-negative mice, as here, it is eGFP^+^ tdTomato^−^. HCMV IE1-driven reporter gene expression reveals acute MCMV infection in a wide range of cell types, including tissue macrophages (23), lymph node macrophages, and dendritic cells (45).

### Virus assays.

To determine the titer of infectious virus, organ homogenates were overlaid onto murine embryonic fibroblasts (4 h, 37°C). The cells were fixed and stained for plaque counting after 4 days. To quantitate viral genomes, DNA from noses was amplified for MCMV genomic coordinates 4166 to 4252 (LightCycler 480 SYBR green; Roche) in parallel with plasmid standards and normalized by β-actin copy number (23).

### Immunostaining.

Noses were fixed in a mixture of 1% formaldehyde, 10 mM sodium periodate, and 75 mM l-lysine (18 h, 4°C), decalcified in phosphate-buffered saline (PBS) containing 250 mM EDTA (3 days), equilibrated in 30% sucrose (18 h), and then frozen in optimal cutting temperature compound (OCT). Sections (6 µm) were air dried (1 h, 23°C), washed three times in PBS, blocked with 0.3% Triton X-100 plus 5% normal goat serum (1 h, 23°C), incubated (18 h, 4°C) with antibodies to olfactory marker protein (goat polyclonal antibody [pAb]; Wako), and eGFP or β-galactosidase (chicken pAb; Abcam), washed three times in PBS, incubated (1 h, 23°C) with Alexa Fluor 568-labeled donkey anti-goat IgG pAb and Alexa Fluor 488-labeled goat anti-chicken IgG pAb (Life Technologies), washed three times in PBS, stained with 4′,6′-diamidino-2-phenylindole (DAPI), and examined with a Zeiss LSM510 confocal microscope.

### ELISA.

MCMV was disrupted with 0.05% Triton X-100, cleared of debris by centrifugation (500 × *g*, 10 min), diluted in 50 mM sodium carbonate (pH 8.5), and coated (18 h, 4°C) onto Maxisorp ELISA plates (Nalge Nunc). Plates were washed three times in PBS containing 0.1% Tween 20 (PBS-T), blocked with PBS-T containing 2% bovine serum albumin, incubated with serum dilutions (1 h, 23°C), washed four times in PBS-T, incubated (1 h, 23°C) with alkaline phosphatase-labeled goat anti-mouse IgG-Fc pAb or alkaline phosphatase-labeled goat anti-mouse IgM-Fc pAb (Southern Biotechnology), washed five times, and developed with nitrophenylphosphate (SpectraMax).

## SUPPLEMENTAL MATERIAL

Figure S1  Infection revealed by viral eGFP expression and lytic antigen staining. (a) Mice were infected i.n. with eGFP plus MCMV (10^5^ PFU in 30 µl under isoflurane anesthesia). One day later, lung sections were stained with a polyclonal goat serum for eGFP (green) and with a polyclonal rabbit serum for MCMV antigens (cyan). Colocalization appears white. Counterstaining was with DAPI. The arrowhead shows an infected cell. All eGFP^+^ cells were also MCMV antigen positive. (b) For a negative control, mice were infected i.n. with eGFP^+^ MuHV-4 (10^5^ PFU in 30 µl under isoflurane anesthesia). One day later, lung sections were stained as described above for panel a. The arrowhead shows an infected cell that is eGFP^+^ and MCMV antigen negative. (c) Mice were infected i.n. with eGFP plus MCMV (10^5^ PFU in 5 µl without anesthesia). Sections of noses taken 3 days later were stained as described above for panel a. The open arrowhead shows a lytically infected neuron. The filled arrowhead shows an eGFP^+^ but MCMV antigen-negative sustentacular cell. (d) Mice were infected and tissue stained for eGFP and MCMV antigens as described above for panel c. The open arrowhead shows an eGFP^+^ lytic antigen-positive subepithelial monocyte/macrophage in the region of the nasal-associated lymphoid tissue. No such infection was seen at 1 day postinfection. Download Figure S1, PDF file, 0.2 MB

Figure S2  Heparan dependence of MCMV infection. EGFP^+^ MCMV, HSV-1, and MuHV-4 were incubated with heparin at various concentrations (2 h, 37°C) and then added to BHK-21 cell monolayers (0.2 PFU/cell), still in the presence of heparin. Eighteen hours later, infection was quantitated by flow cytometric assay of eGFP expression. Bars show means ± standard deviations (SD) of triplicate infections, each expressed as a percentage of the no-heparin control (20 to 50% of total cells eGFP^+^). The inhibition of each infection by heparin was highly significant (*P* < 10^−6^ by χ^2^ test, comparing infected and uninfected populations). Download Figure S2, PDF file, 0.1 MB

Figure S3  MCMV transmission between pups (see experiment 3 in Fig. 6). (a) BALB/c pups given M78-LUC MCMV i.n. (three *in vivo* infectious doses) were tracked by live imaging. An early presentation (day 8) with nasal infection and a later presentation (day 17) with disseminated infection are shown. Each panel shows an inoculated mouse and an uninoculated control. The abdominal signals in the left-hand panel are background light emission from the liver. (b) Examples of weak positive live image signals (arrowheads) of recipient pups cohoused with infected donors. The top left mouse is a nonexposed control. (c) Dissection of tissues from an infected recipient mouse, showing luciferase signal in the nose (arrowhead) but not other organs. (d) Nasal signal of an infected recipient pup (arrowhead) and three uninfected controls. Download Figure S3, PDF file, 0.8 MB

Figure S4  MCMV transmission from parents to pups (see experiment 4 in Fig. 6a). (a) Male and female adult BALB/c mice were infected i.n. with M78-LUC MCMV (10^5^ PFU without anesthesia) and then mated (left-hand panel). By the time of pregnancy (obvious in the middle mouse) approximately 1 month later, live image luciferase signals were restricted to the salivary glands. Signals were generally higher in females (left-hand mice) than in males (right-hand mouse). All were substantially lower than in the donor pups in experiment 3 in Fig. 6, as shown in the right-hand panel. Note the difference in scales. (b) Weak positive live image signals of pups with MCMV-infected parents (exposed, arrowheads), each compared with a control pup of uninfected parents. (c) Dissection of noses showing weak positive signal in a pup with MCMV-infected parents (arrowhead) compared with noses of 2 control pups with uninfected parents. (d) Dissection of a pup with infected parents, showing weak positive signal in the nose and not in other organs. Download Figure S4, PDF file, 0.8 MB
