# Supplementary material for: Murine Cytomegalovirus Exploits Olfaction To Enter New Hosts
Source: mBio. 2016 Apr 26;7(2):e00251-16. doi: 10.1128/mBio.00251-16 (PMC4850257; doi:10.1128/mBio.00251-16)
Supplement: Figure S1 — Infection revealed by viral eGFP expression and lytic antigen staining. (a) Mice were infected i.n. with eGFP plus MCMV (105 PFU in 30 µl under isoflurane anesthesia). One day later, lung sections were stained with a polyclonal goat serum for eGFP (green) and with a polyclonal rabbit serum for MCMV antigens (cyan). Colocalization appears white. Counterstaining was with DAPI. The arrowhead shows an infected cell. All eGFP+ cells were also MCMV antigen positive. (b) For a negative control, mice were infected i.n. with eGFP+ MuHV-4 (105 PFU in 30 µl under isoflurane anesthesia). One day later, lung sections were stained as described above for panel a. The arrowhead shows an infected cell that is eGFP+ and MCMV antigen negative. (c) Mice were infected i.n. with eGFP plus MCMV (105 PFU in 5 µl without anesthesia). Sections of noses taken 3 days later were stained as described above for panel a. The open arrowhead shows a lytically infected neuron. The filled arrowhead shows an eGFP+ but MCMV antigen-negative sustentacular cell. (d) Mice were infected and tissue stained for eGFP and MCMV antigens as described above for panel c. The open arrowhead shows an eGFP+ lytic antigen-positive subepithelial monocyte/macrophage in the region of the nasal-associated lymphoid tissue. No such infection was seen at 1 day postinfection. Download [file mbo002162790sf1.pdf]

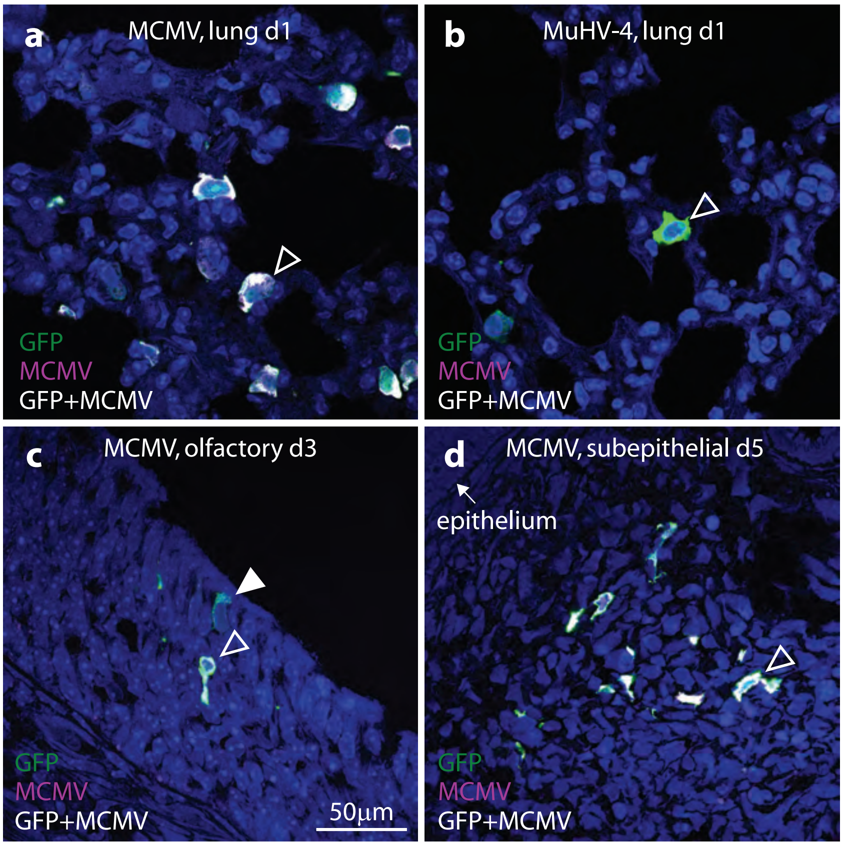

Figure S1. Infection revealed by viral eGFP expression and lytic antigen staining.

a. Mice were infected i.n. with eGFP^{+} MCMV (10^{5} p.f.u. in 30 l under isoflurane anesthesia). 1d later lung sections were stained for eGFP (green) with a polyclonal goat serum and for MCMV antigens (cyan) with a polyclonal rabbit serum. Co-localization appears white. Counterstaining was with DAPI. The arrow shows an example infected cell. All eGFP^{+} cells werealso MCMV antigen^{+}.

b. As a negative control, mice were infected i.n. with eGFP^{+} MuHV-4 (10^{5} p.f.u. in 30 l under isoflurane anesthesia). 1d later lung sections were stained as in a. The arrow shows an infected cell that is eGFP^{+} and MCMV antigen^{-}.

c. Mice were infected i.n. with eGFP^{+} MCMV (10^{5} p.f.u. in 5 l without anesthesia). Sections of noses taken 3d later were stained as in **a**. The open arrow shows a lytically infected neuron. The filled arrow shows an eGFP^{+} but MCMV antigen^{-} sustentacular cell.

d. Mice were infected and tissue stained for eGFP and MCMV antigens as in **c**. The open arrow shows an eGFP^{+}lytic antigen^{+} subepithelial monocyte/macrophage in the region of the nasal-associated lymphoid tissue. No such infection was seen at 1d post-infection.
