# Supplementary material for: Murine Cytomegalovirus Exploits Olfaction To Enter New Hosts
Source: mBio. 2016 Apr 26;7(2):e00251-16. doi: 10.1128/mBio.00251-16 (PMC4850257; doi:10.1128/mBio.00251-16)
Supplement: Figure S2 — Heparan dependence of MCMV infection. EGFP+ MCMV, HSV-1, and MuHV-4 were incubated with heparin at various concentrations (2 h, 37°C) and then added to BHK-21 cell monolayers (0.2 PFU/cell), still in the presence of heparin. Eighteen hours later, infection was quantitated by flow cytometric assay of eGFP expression. Bars show means ± standard deviations (SD) of triplicate infections, each expressed as a percentage of the no-heparin control (20 to 50% of total cells eGFP+). The inhibition of each infection by heparin was highly significant (P < 10−6 by χ2 test, comparing infected and uninfected populations). Download [file mbo002162790sf2.pdf]

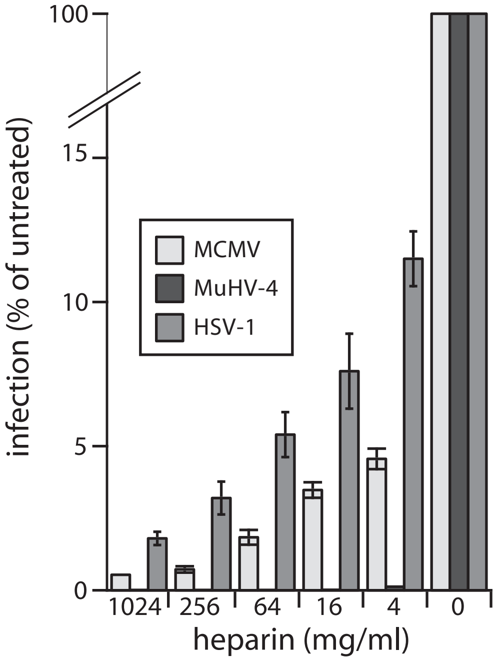

Figure S2. Heparan dependence of MCMV infection.
EGFP+ MCMV. HSV-1 and MuHV-4 were incubated with heparin at varying concentration (2h, 37°C) then added to BHK-21 cell monolayers (0.2 p.f.u. / cell), still in the presence of heparin. 18h later infection was quantitated by flow cytometric assay of eGFP expression. Bars show mean ± SD of triplicate infections, each expressed as a percentage of the no heparin control (20-50% of total cells eGFP+). The inhibition of each infection by heparin was highly significant ($p<10^{-6}$ by X^2 test, comparing infected and uninfected populations).
