# Supplementary material for: Murine Cytomegalovirus Exploits Olfaction To Enter New Hosts
Source: mBio. 2016 Apr 26;7(2):e00251-16. doi: 10.1128/mBio.00251-16 (PMC4850257; doi:10.1128/mBio.00251-16)
Supplement: Figure S3 — MCMV transmission between pups (see experiment 3 in Fig. 6). (a) BALB/c pups given M78-LUC MCMV i.n. (three in vivo infectious doses) were tracked by live imaging. An early presentation (day 8) with nasal infection and a later presentation (day 17) with disseminated infection are shown. Each panel shows an inoculated mouse and an uninoculated control. The abdominal signals in the left-hand panel are background light emission from the liver. (b) Examples of weak positive live image signals (arrowheads) of recipient pups cohoused with infected donors. The top left mouse is a nonexposed control. (c) Dissection of tissues from an infected recipient mouse, showing luciferase signal in the nose (arrowhead) but not other organs. (d) Nasal signal of an infected recipient pup (arrowhead) and three uninfected controls. Download [file mbo002162790sf3.pdf]

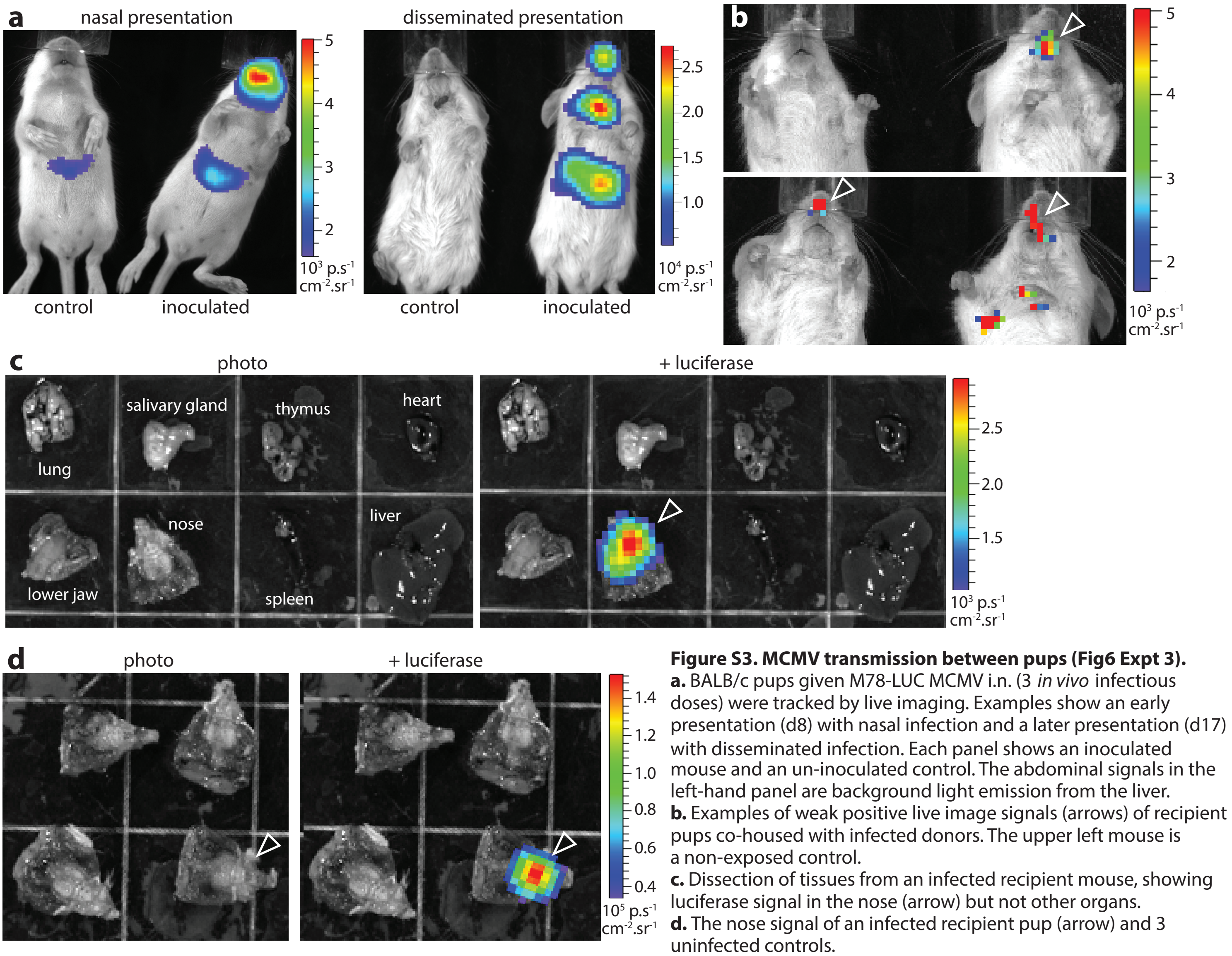

Figure S3. MCMV transmission between pups (Fig6 Expt 3).
a. BALB/c pups given M78-LUC MCMV i.n. (3 *in vivo* infectious doses) were tracked by live imaging. Examples show an early presentation (d8) with nasal infection and a later presentation (d17) with disseminated infection. Each panel shows an inoculated mouse and an un-inoculated control. The abdominal signals in the left-hand panel are background light emission from the liver.
b. Examples of weak positive live image signals (arrows) of recipient pups co-housed with infected donors. The upper left mouse is a non-exposed control.
c. Dissection of tissues from an infected recipient mouse, showing luciferase signal in the nose (arrow) but not other organs.
d. The nose signal of an infected recipient pup (arrow) and 3 uninfected controls.
