# Supplementary material for: Murine Cytomegalovirus Exploits Olfaction To Enter New Hosts
Source: mBio. 2016 Apr 26;7(2):e00251-16. doi: 10.1128/mBio.00251-16 (PMC4850257; doi:10.1128/mBio.00251-16)
Supplement: Figure S4 — MCMV transmission from parents to pups (see experiment 4 in Fig. 6a). (a) Male and female adult BALB/c mice were infected i.n. with M78-LUC MCMV (105 PFU without anesthesia) and then mated (left-hand panel). By the time of pregnancy (obvious in the middle mouse) approximately 1 month later, live image luciferase signals were restricted to the salivary glands. Signals were generally higher in females (left-hand mice) than in males (right-hand mouse). All were substantially lower than in the donor pups in experiment 3 in Fig. 6, as shown in the right-hand panel. Note the difference in scales. (b) Weak positive live image signals of pups with MCMV-infected parents (exposed, arrowheads), each compared with a control pup of uninfected parents. (c) Dissection of noses showing weak positive signal in a pup with MCMV-infected parents (arrowhead) compared with noses of 2 control pups with uninfected parents. (d) Dissection of a pup with infected parents, showing weak positive signal in the nose and not in other organs. Download [file mbo002162790sf4.pdf]

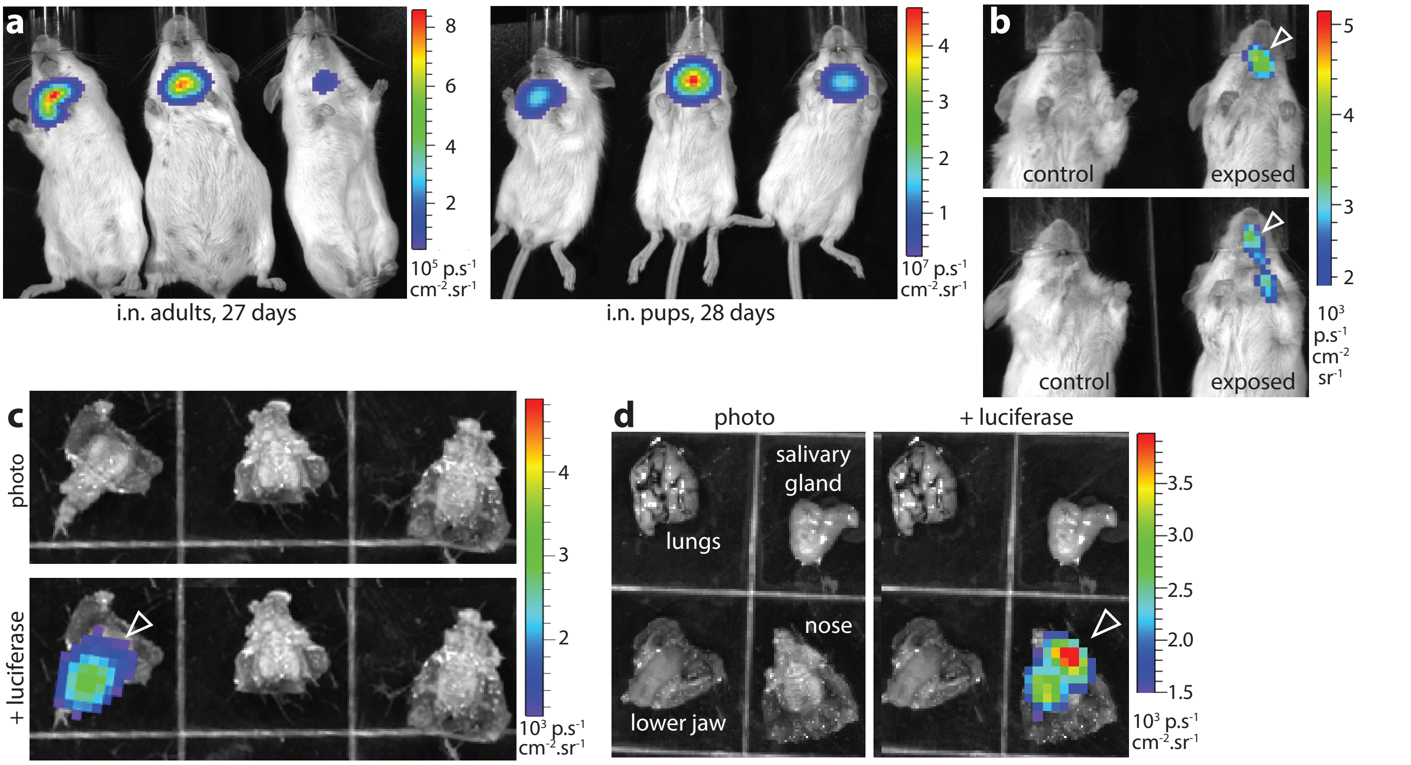

Figure S4. MCMV transmission from parents to pups (Fig 6a Expt 4).

a. Male and female adult BALB/c mice were infected i.n. with M78-LUC MCMV (10^5 p.f.u. without anesthesia) then mated (left hand panel). By the time of pregnancy (obvious in the middle mouse) approximately 1 month later, live image luciferase signals were restricted to the salivary glands. Signals were generally higher in females (left-hand mice) than in males (right-hand mouse). All were substantially lower than in the donor pups of Fig.6 Expt 3, as shown in the right-hand panel. Note the different scales.

b. Example weak positive live image signals of pups with MCMV-infected parents (exposed, arrows), each compared with a control pup of uninfected parents.

c. Dissection of noses showing weak positive signal in an example pup with MCMV-infected parents (arrow) compared with noses of 2 control pups with uninfected parents.

d. Dissection of an example pup with infected parents, showing weak positive signal in the nose and not in other organs.
